# Validation of the SSTR-RADS 1.0 for the structured interpretation of SSTR-PET/CT and treatment planning in neuroendocrine tumor (NET) patients

**DOI:** 10.1007/s00330-023-09518-y

**Published:** 2023-03-25

**Authors:** Freba Grawe, Ricarda Ebner, Thomas Geyer, Leonie Beyer, Michael Winkelmann, Gabriel T. Sheikh, Ralf Eschbach, Christine Schmid-Tannwald, Clemens C. Cyran, Jens Ricke, Peter Bartenstein, Maurice M. Heimer, Lorenzo Faggioni, Christine Spitzweg, Matthias P. Fabritius, Christoph J. Auernhammer, Johannes Ruebenthaler

**Affiliations:** 1 Department of Radiology, University Hospital, LMU Munich, Marchioninistr. 15, 81377 Munich, Germany; 2 Department of Nuclear Medicine, University Hospital, LMU Munich, 81377 Munich, Germany; 3 Department of Internal Medicine 4, University Hospital, LMU Munich, 81377 Munich, Germany; 4 Interdisciplinary Center of Neuroendocrine Tumors of the GastroEnteroPancreatic System (GEPNET-KUM, ENETS Certified Center of Excellence), University Hospital, LMU Munich, 81377 Munich, Germany; 5 Department of Translational Research, Academic Radiology, University of Pisa, Via Roma, 67, 56126 Pisa, Italy

**Keywords:** Neuroendocrine tumors, Positron emission tomography computed tomography, Somatostatin

## Abstract

**Objectives:**

The recently proposed standardized reporting and data system for somatostatin receptor (SSTR)–targeted PET/CT SSTR-RADS 1.0 showed promising first results in the assessment of diagnosis and treatment planning with peptide receptor radionuclide therapy (PRRT) in neuroendocrine tumors (NET). This study aimed to determine the intra- and interreader agreement of SSTR-RADS 1.0.

**Methods:**

SSTR-PET/CT scans of 100 patients were independently evaluated by 4 readers with different levels of expertise according to the SSTR-RADS 1.0 criteria at 2 time points within 6 weeks. For each scan, a maximum of five target lesions were freely chosen by each reader (not more than three lesions per organ) and stratified according to the SSTR-RADS 1.0 criteria. Overall scan score and binary decision on PRRT were assessed. Intra- and interreader agreement was determined using the intraclass correlation coefficient (ICC).

**Results:**

Interreader agreement using SSTR-RADS 1.0 for identical target lesions (ICC ≥ 0.91) and overall scan score (ICC ≥ 0.93) was excellent. The decision to state “functional imaging fulfills requirements for PRRT and qualifies patient as potential candidate for PRRT” also demonstrated excellent agreement among all readers (ICC ≥ 0.86). Intrareader agreement was excellent even among different experience levels when comparing target lesion–based scores (ICC ≥ 0.98), overall scan score (ICC ≥ 0.93), and decision for PRRT (ICC ≥ 0.88).

**Conclusion:**

SSTR-RADS 1.0 represents a highly reproducible and accurate system for stratifying SSTR-targeted PET/CT scans with high intra- and interreader agreement. The system is a promising approach to standardize the diagnosis and treatment planning in NET patients.

**Key Points:**

*• SSTR-RADS 1.0 offers high reproducibility and accuracy.*

*• SSTR-RADS 1.0 is a promising method to standardize diagnosis and treatment planning for patients with NET.*

**Supplementary Information:**

The online version contains supplementary material available at 10.1007/s00330-023-09518-y.

## Introduction

Standardized interpretation in oncological imaging has gained increasing importance as it provides reproducible and consistent reports, facilitates communication with the referring clinician, and minimizes misinterpretation of imaging pitfalls [1–3]. Numerous Reporting and Data Systems (RADS) have been established for different organs and diagnostic modalities such as LI-RADS for hepatocellular carcinoma in MRI and CT; BI-RADS for breast lesions in mammography, ultrasound, and MRI; or PI-RADS for prostate cancer in MRI (https://www.acr.org/Clinical-Resources/Reporting-and-Data-Systems). For patients with well-differentiated neuroendocrine tumors (NET), a novel standardized framework for the interpretation of somatostatin receptor (SSTR)–positron emission tomography/computed tomography (PET/CT) has been introduced titled SSTR-RADS 1.0 [3]. SSTR are overexpressed in the cell membrane of NET, which forms the basis for the affinity of radiolabeled SSTR analogs [4, 5]. SSTR expression makes NET lesions accessible not only for functional imaging but also for targeted therapy (peptide receptor radionuclide therapy, PRRT), which is a systemic treatment option in inoperable, metastatic NET patients. The extent of SSTR expression in PET/CT indicates the patients’ eligibility for treatment [6]. Large studies, such as the NETTER-1 trial, demonstrated that PRRT significantly prolongs the progression-free survival and the time to health-related quality-of-life deterioration and showed a clinically meaningful (but not significant) increase of overall survival of 11.7 months [7–9]. In the evaluation of SSTR-PET/CT scans, the SSTR expression of NET lesions has been reported descriptively so far, mainly based on the ratio of SSTR uptake in the liver compared to the tumor, the so-called Krenning’s score [10–12]. The proposed standardized reporting and data system SSTR-RADS 1.0 for SSTR-PET/CT showed promising first results in the assessment of diagnosis and treatment planning with PRRT in patients with NET [13, 14]. This study aims to determine the interreader agreement of four readers with different experience levels and the intrareader agreement in a second read 6 weeks later using SSTR-RADS 1.0 to further assess the feasibility of the proposed framework in routine clinical practice.

## Methods

### Study patients

For this retrospective study, patients were selected from an institutional database with histologically confirmed or suspected NET who underwent SSTR-PET/CT between April and November 2020. Only patients who received the mainly used tracer DOTA(0)-Phe(1)-Tyr(3))octreotide (DOTA-TOC) at inclusion time were selected. Patients receiving other tracers than DOTA-TOC were excluded for homogeneity reasons. Imaging was performed for initial staging or follow-up examination. Further inclusion criteria were complete clinical and imaging data. Patient characteristics are presented in Table 1. Almost all patients were pretreated (*n* = 95) at the time of the reading depending on numerous factors such as tumor grading, size and site of the primary tumor, Ki-67, and presence of metastases. Therapy included surgery, somatostatin analogs, chemotherapy, and locoregional procedures either as single therapy or in combination. Patients who underwent PRRT before PET/CT imaging were excluded.

**Table 1:** Patient characteristics

Characteristics		*N* = 100
Age (mean ± SD)	61 ± 15 y	
Sex	Female/male	53/47
Indication for SSTR-PET/CT	Staging/re-staging	21/79
Grading	(Not available in 3 patients)	
	G1	39
	G2	52
	G3	6
Primary tumor	GEP-NET	92
	Ileum/jejunum/mesenterial	58
	Pancreas	31
	Rectum	2
	Colon	1
	Non-GEP-NET	8
	Lung	4
	CUP (no primary tumor was detectable)	2
	Others	2
Prior therapies	Patients pretreated	95
	Surgery	65
	Somatostatin analog	55
	Chemotherapy	24
	Locoregional procedure	12
	Tyrosine kinase inhibitor (TKI)	5

*SD* standard deviation, *SSTR* somatostatin receptor, *G* grade, *GEP* gastroenteropancreatic, *NET* neuroendocrine tumor, *CUP* cancer of unknown primary

### DOTA-TOC-PET/CT imaging

SSTR-PET/CT scans were acquired on Biograph 64 TruePoint w/TrueV and Biograph mCT Flow 20-4R PET/CT scanners (Siemens, Healthcare GmbH) and were acquired approximately 60 min after intravenous administration of 232 ± 36 MBq radiolabelled somatostatin analogs (^68^Ga-DOTA-TOC). After intravenous injection of contrast agent (*n* = 96; Ultravist 300, Bayer Vital GmbH or Imeron 350 mgl/mL, 2.5 mL/s, Bracco Imaging), diagnostic CT scans of the neck, thorax, abdomen, and pelvis (100–190 mAs; 120 kV) were acquired. Patients received diagnostic CT scans without contrast enhancement (*n* = 4) in case of known allergic reactions to iodinated contrast agent, renal impairment/failure, or hyperthyroidism. Image analysis was performed using a dedicated software package (Hermes Hybrid Viewer, Hermes Medical Solutions). All acquired PET/CT scans were analyzed using dedicated software packages (syngo.via, Siemens Healthcare or Hermes Hybrid Viewer, Hermes Medical Solutions).

### Readers

The PET/CT scans of all 100 included study patients (one per person) were evaluated by a board-certified radiologist and nuclear medicine physician (experienced reader (ER) 1 and 2,  > 7 years of experience in PET/CT imaging), respectively, as well as one radiology and one nuclear medicine resident (inexperienced reader (IR) 1 and 2,  < 2 years of experience in PET/CT imaging), respectively. Readers were masked to the clinical patient data except age and sex of the patient. All readers were familiar with the used workstations and software from clinical routine and were introduced to the SSTR-RADS 1.0 before the first read.

### SSTR-RADS version 1.0 and image interpretation

Lesions classified as SSTR-RADS 1 are definitely benign. SSTR-RADS 2 defines lesions with a minor level of SSTR expression or non-specific radiotracer uptake at an atypical site for NET, indicating that the lesions are almost certainly benign. Further workup (subsequent biopsy or follow-up imaging) is required for SSTR-RADS 3 lesions. These imaging findings are suggestive of, but not definitive for, NET. SSTR-RADS 4 includes those findings having an enhanced SSTR expression in sites typical for NET lesions, but without definitive findings on conventional imaging, whereas SSTR-RADS 5 shows intense uptake in sites typical for NET lesions with corresponding findings on conventional imaging. A detailed overview of the SSTR-RADS 1.0 is described in the original work [3].

For the evaluation of interreader agreement, all four readers were encouraged to choose a maximum of five target lesions (TLs) for each scan, with no more than three of the five TLs assigned to the same compartment. The imaging findings that are most apparent in CT imaging or have the highest tracer uptake on PET should be included in the selection. Predefined organ compartments were liver, lymph nodes (LNs), soft tissue (other than LNs), skeleton, and lung. An overall scan score was determined, which corresponded to the highest SSTR-RADS score of all individual TLs. After each TL was assigned to one SSTR-RADS score, the readers decided whether a PRRT was reasonable for the patient based on the assigned scores and the general image impression. In order to be able to evaluate a higher number of lesions and to determine intrareader agreement, all scans were examined a second time 6 weeks after the first read by the four readers under the same conditions.

### Statistical analysis

Continuous variables were expressed as mean ± SD and categorical variables as *N* (%). The agreement of SSTR-RADS 1.0 was evaluated using the intraclass correlation coefficient (ICC) and their 95% CIs. For the analysis of intra- and interreader agreement, Shrout & Fleiss ICC (2,1) was used. According to Cicchetti, ICC values  < 0.40 indicate poor agreement, 0.40–0.59 indicate fair agreement, 0.60–0.74 were considered as good, and  ≥ 0.75 were considered as excellent [15]. A *p* value  < 0.05 was considered statistically significant. All analyses were performed using SPSS computer software (SPSS Statistics 25, IBM). ICC agreements between two groups were compared with “cocron” [16].

## Results

### Interreader agreement for compartments

A total of 3037 TL were chosen by all 4 readers. Of these, 1058 TLs were selected at least once. Identical TLs were selected by all four readers in 127 cases in the first read and in 115 cases in the second read. The distribution of the TLs among the compartments is shown in the supplementary material in Table S1.

The interreader agreement for scoring identically chosen TLs by all four readers was excellent with an ICC of 89% in the first read and 91% in the second read. Even when evaluated separately for readers classified by their level of experience, the interreader agreement showed excellent results with an ICC of 92% in the first read and 91% in the second read for ERs and 83% in the first and 82% in the second read for IRs.

In the compartment-based analysis, excellent results could be found among most organs with ICCs  ≥ 76% for both reads as presented in Table 2. LN scoring according to SSTR-RADS resulted in an ICC of 76% in the first read and only 50% in the second read.

**Table 2:** Interreader agreement of SSTR-RADS for 4 identical target lesions (TL) among all 4 readers regarding reader types and organ system

Inter-reader agreement ICC [95% CI]	Reader type		Organ system					All readers all organs
	ER	IR	Liver	LN	Soft tissue	Skeleton	Lung	
1^st^ read	0.924 [0.899; 0.942]	0.831 [0.779; 0.871]	0.900 [0.840; 0.942]	0.756 [0.585; 0.868]	0.992 [0.960; 0.999]	0.778 [0.513; 0.917]	0.916 [0.856; 0.954]	0.892 [0.858; 0.920]
2^nd^ read	0.914 [0.884; 0.936]	0.819 [0.758; 0.865]	0.937 [0.899; 0.963]	0.501 [0.101; 0.749]	0.944 [0.902; 0.971]	0.772 [0.470; 0.922]	1.000 [1.000; 1.000]	0.909 [0.879; 0.934]

*ICC* intraclass correlation coefficient, *CI* confidence interval, *ER* experienced reader, *IR* inexperienced reader, *LN* lymph nodes

### Interreader agreement for the overall scan score

**Fig. 1:**
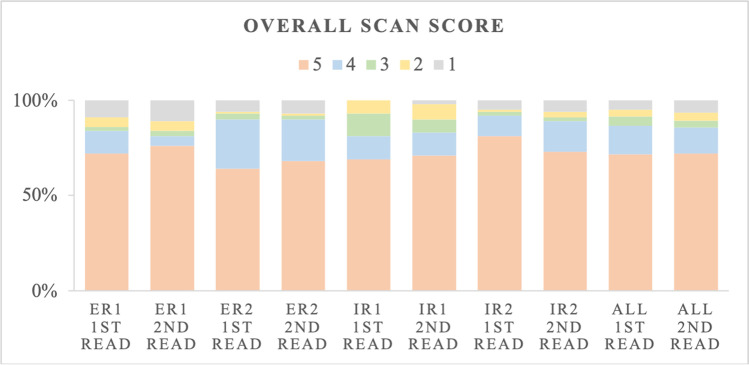
Distribution of SSTR-RADS for the overall scan score of experienced (ER) and inexperienced readers (IR)

In the evaluation of the overall scan score, the ICC for all four readers was 91% in the first read and 93% in the second read. Even among IRs, ICC was excellent with 87% in the first read and 85% in the second read as seen in Table 3. From the 100 evaluated SSTR-PET/CT scans, most of the scans were rated as SSTR-RADS score 4 or 5 by all four readers as presented in Fig. 1. Dedicated results are presented in the supplements in Table S2.

**Table 3:** Interreader agreement for the overall scan score among experienced (ER) and inexperienced readers (IR)

Interreader agreement ICC [95% CI]	Overall scan score		
	ER	IR	All readers
1^st^ read	0.864 [0.797; 0.908]	0.873 [0.812; 0.915]	0.914 [0.883; 0.939]
2^nd^ read	0.869 [0.805; 0.912]	0.847 [0.773; 0.897]	0.925 [0.897; 0.946]

*ICC* intraclass correlation coefficient, *CI* confidence interval

### Interreader agreement for treatment decision with PRRT

**Fig. 2:**
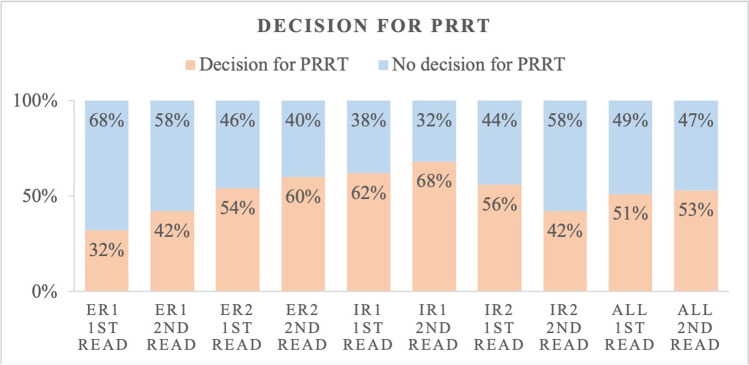
Treatment decision “functional imaging fulfils requirements for PRRT and qualifies patient as potential candidate for peptide receptor radionuclide therapy (PRRT)” among experienced (ER) and inexperienced readers (IR)

All 4 readers were asked whether they would consider PRRT for each patient based on the assigned SSTR-RADS scores and the general image impression. Among ERs, excellent results were achieved for the recommendation of PRRT in both reads (ICC 77% and 79%; Table 4). Among IRs, the agreement was good in the first read (ICC 68%) as well as in the second read (ICC 66%). The overall agreement on treatment decision was high in both reads (ICC 81% and 86%). However, among all 4 readers, IRs decided more frequently on PRRT in both reads (*n* = 228) compared to ERs (*n* = 188) as illustrated in Fig. 2.

**Table 4:** Interreader agreement on the decision for peptide receptor radionuclide therapy (PRRT) among experienced (ER) and inexperienced readers (IR)

Interreader agreement ICC [95% CI]	Decision for PRRT		
	ER	IR	All readers
1^st^ read	0.774 [0.665; 0.848]	0.675 [0.517; 0.781]	0.811 [0.742; 0.865]
2^nd^ read	0.790 [0.688; 0.859]	0.663 [0.499; 0.773]	0.864 [0.815; 0.903]

*ICC* intraclass correlation coefficient, *CI* confidence interval

### Intrareader agreement for compartments, overall scan score, and decision for PRRT

Intrareader agreement was excellent among ER and IR for the scoring of compartments and the overall scan score with ICCs  ≥ 92%.

**Fig. 3:**
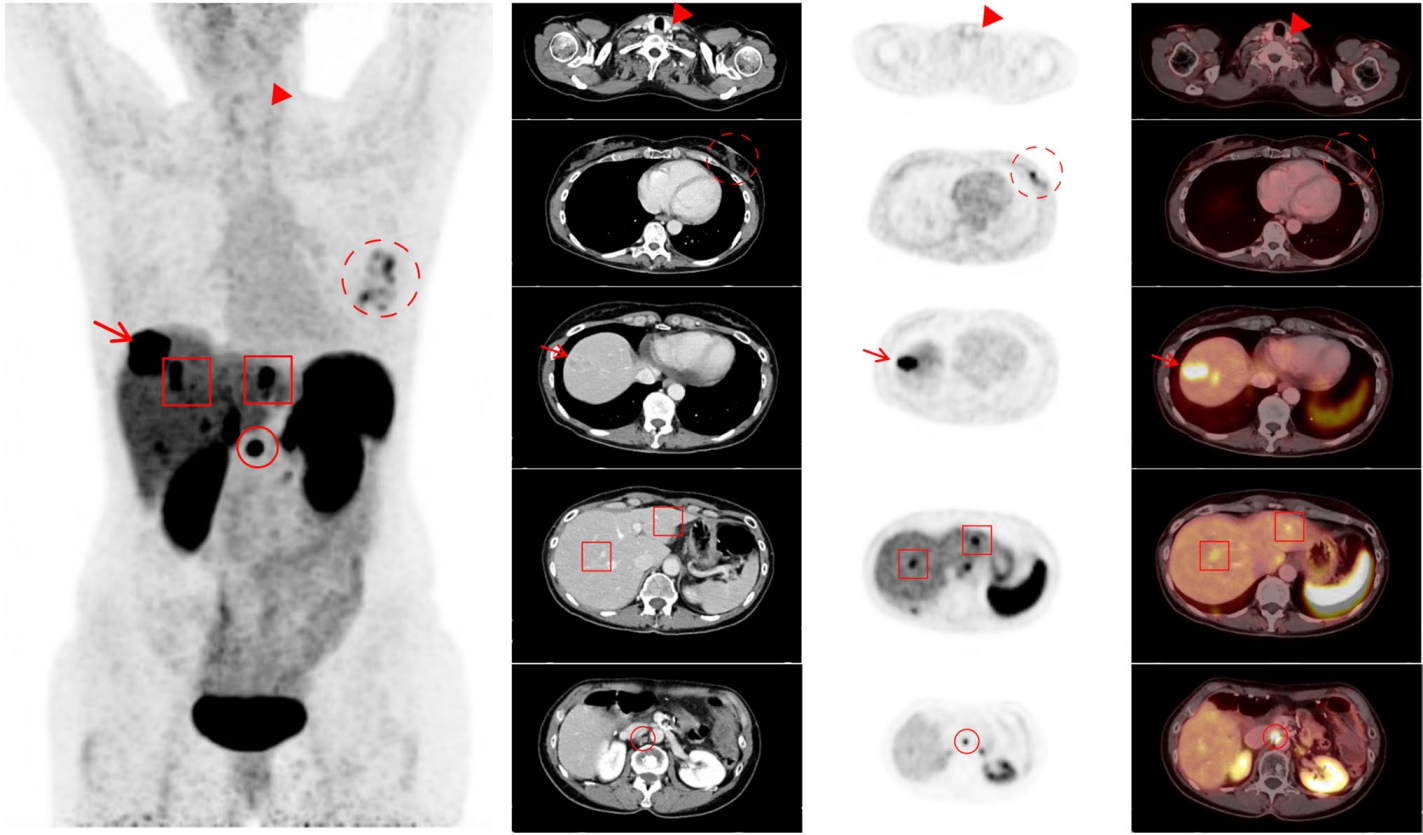
50-year-old woman with neuroendocrine tumor of the pancreas. The patient underwent contrast-enhanced diagnostic CT. The thyroid gland was defined as SSTR-RADS 1, with no abnormal tracer uptake. Axial CT, ^68^ Ga-DOTATOC PET, and fused PET/CT show a visible lesion with moderate tracer uptake (dashed circle) in the left breast compatible with fibroadenoma. SSTR-RADS 3C was assigned by all readers except one ER in one read. There is intense focal uptake in the liver dome (arrow) with corresponding finding on CT. This lesion was classified as SSTR-RADS-5 by all 4 readers. However, there are more lesions (red square) in segment II and VIII with no corresponding finding on CT (assigned SSTR-RADS 4 by only 2 readers in both reads). Intense uptake in a mesenterial lymph node can be noted (red circle). All readers identified the corresponding finding on CT, so this lesion was classified as SSTR-RADS-5 by all readers. All 4 readers except one ER in one read recommended PRRT according to the SSTR-RADS 1.0 criteria in this patient

For the decision of treatment with PRRT, slightly lower ICC values were observed, with an ICC of 87% for ERs, 89% for IRs, and 88% for all 4 readers (Table 5). A patient example with assigned SSTR-RADS scores is presented in Fig. 3.

**Table 5:** Intrareader agreement on organ system–/target lesion–based, overall scan score and decision for peptide receptor radionuclide therapy (PRRT) scoring among experienced (ER) and inexperienced readers (IR)

	Reader type		Organ system					All readers All organs
	ER	IR	Liver	LN	Soft tissue	Skeleton	Lung	
Intra-reader agreement ICC [95% CI]	0.976 [0.971; 0.980]	0.989 [0.987; 0.990]	0.983 [0.979; 0.987]	0.950 [0.935; 0.961]	0.983 [0.980; 0.986]	0.942 [0.915; 0.961]	0.979 [0.957; 0.990]	0.983 [0.980; 0.985]
Intrareader agreement ICC [95% CI]		Overall scan score						
		ER		IR		All readers		
		0.925 [0.901; 0.943]		0.924 [0.899; 0.942]		0.925 [0.908; 0.938]		
Intrareader agreement ICC [95% CI]		Decision for PRRT						
		ER		IR		All readers		
		0.870 [0.829; 0.902]		0.888 [0.852; 0.915]		0.877 [0.850; 0.899]		

*ICC* intraclass correlation coefficient, *CI* confidence interval, *LN* lymph nodes

## Discussion

A novel framework for the standardized interpretation of SSTR-PET/CT and treatment planning of NET patients has been introduced in analogy to previously established RADS, titled SSTR-RADS version 1.0 [3]. The present multireader study was conducted to validate the reader-dependent reproducibility of the standardized reporting system SSTR-RADS. SSTR-RADS was applied to SSTR-PET/CT scans of 100 NET patients by readers with low and high levels of experience to evaluate interreader agreement. Moreover, SSTR-PET/CT scans were presented a second time to the readers after 6 weeks to assess intrareader agreement.

Applying the SSTR-RADS score to the SSTR-PET/CT scans, an overall excellent inter- and intrareader agreement was observed for the overall scan score in both the first and the second reads. These results were consistent among readers with different levels of experience, confirming high reproducibility of SSTR-RADS and simple application even for inexperienced readers, which is essential to provide the clinician with reliable information. Our results are in line with previously published studies by Fendler et al and Werner et al who reported on ICCs  ≥ 85% in the assessment of the overall scan score [14, 17].

Since the theranostic approach for NET has developed into a standardized diagnostic and therapeutic procedure in recent years, accurate assessment of the overall scan score is of utmost importance for selecting eligible patients for PRRT [6, 12, 18–20]. Our analysis showed that less experienced readers considered PRRT overall more often (*n* = 228) than experienced readers (*n* = 188), which underlines the findings from previously published data. Fendler et al reported that IRs considered inappropriately more frequent PRRT compared to ERs, and therefore recommended interpretation of SSTR-PET/CT scans by ERs in this case [21]. In contrast to our study, Fendler et al referred to the primary nuclear medicine physician as the reference standard who had access to all clinical data, which was not the case in our study. Werner et al reported significantly varying results for considering PRRT among ERs and IRs, which further emphasizes our discrepant findings. However, even though decision-making for PRRT seems to require experience and training, the overall interreader agreement among all four readers was excellent in both reads. Therefore, the present study confirms that the proposed framework system should be considered for implementation into clinical routine as SSTR-RADS seems to serve as a guide for nuclear medicine physicians in the consideration of PRRT.

Currently, Krenning’s score is most commonly used but novel molecular imaging reporting and data system (MI-RADS) as the SSTR-RADS 1.0 score might be promising. However, further clinical studies are required to evaluate the clinical outcome of patient selection for PRRT based on SSTR-RADS 1.0 score. An appropriate scan score is of utmost importance for selecting eligible patients for PRRT [12, 20] and the nuclear medicine physicians’ general statement “functional imaging fulfills requirements for PRRT and qualifies patient as potential candidate for PRRT.” Moreover, in all cases PRRT is considered, double reading should be implemented by a senior physician. However, a definite treatment decision for PRRT in a theranostic center requires clinical case discussion in a multidisciplinary team (MDT) [20], and MDTs are considered a quality performance indicator [22]. The MDT board discussion should include multiple parameters such as patient history, tumor load, tumor dynamic, primary tumor location, tumor grading, and alternative systemic and local treatment options and thus provide a profound basis for a MDT decision.

Roughly one third of identical TLs were chosen by all four readers. The compartment-based assessment of the SSTR-RADS scoring to SSTR-PET/CT scans mostly showed almost perfect interreader agreements among all readers. In the assessment of LN scoring, the interreader agreement varied between excellent (ICC 76%) in the first read and fair (ICC 50%) in the second read. This finding can be explained by scoring mostly LNs with 4 or 5 but in different numbers. Although these results have statistical impact, in the clinical aspect, both lead to the consideration of PRRT according to SSTR-RADS. Moreover, this finding further emphasizes the relevance of functional imaging especially in evaluating small target lesions such as lymph nodes which can be overseen in anatomical imaging (CT). Based on these results, it seems reasonable to use the SSTR-RADS to describe single lesions from the SSTR-PET/CT findings, assuming there was mostly excellent agreement not just for the overall scan score but also for single lesions. However, since LN scoring showed difficulties, we support the proposal of Werner et al to select TLs stricter and more standardized [14] to further improve SSTR-RADS scoring by, e.g., selecting loco-regional lymph nodes.

Since SSTR-PET/CT plays an increasingly important role in the diagnosis of NETs, such as ^68^Ga-DOTA-TOC-PET/CT for diagnosing and staging of pancreatic NET, and given the increasing availability of PET/CT, several pitfalls in the interpretation of SSTR-PET/CT have been reported in recent years, such as the potential physiological distribution of SSTR on the cell surface of the pituitary gland or adrenal gland and macrophages in the case of inflammation [1–3, 23]. Minimizing these pitfalls is expected to be another characteristic of the SSTR-directed framework. A study by Weich et al showed that aiding interpretation of SSTR-RADS image findings led to reduced anxiety especially in inexperienced readers and increased readers’ confidence [13]. Moreover, the study reported on high motivation to learn such standardized framework and complement it into clinical routine.

The readers received a brief introduction to the SSTR-RADS before the study was conducted. Due to the simplicity and good comprehensibility of the SSTR-RADS, the readers were able to familiarize themselves with the SSTR-RADS in a very short time. Since the 5-point scale SSTR-RADS is structured in a reciprocal fashion with PSMA-RADS for the interpretation of PSMA-PET/CT, both frameworks are summarized under the term molecular imaging (MI)–RADS and can be apparently implemented into clinical routine without significant additional effort [24, 25].

There are a few limitations of this study. First, no histopathological comparison was available to validate each TL. Second, all readers were blinded to the clinical status of the study patients, which may have reduced interreader agreement and, with a better understanding of the clinical situation, interreader agreement may increase even further. Further studies could also evaluate the performance of inexperienced readers against a reference standard established by a consensus interpretation of several experienced readers or by an experienced reader provided with all clinical information. In conclusion, SSTR-RADS 1.0 represents a highly reproducible and accurate system for stratifying SSTR-targeted PET/CT imaging in NET patients with high inter- and intrareader agreement among readers with different levels of experience. The proposed scoring system represents a useful tool for simplifying and improving the management of NET patients in clinical practice by the standardization of diagnosis and treatment planning. However, in the compartment-based assessment of the SSTR-RADS score, lymph nodes should be carefully selected and scored. Furthermore, image-based decisions on PRRT should be taken by rather experienced physicians.

## Supplementary Information

Below is the link to the electronic supplementary material.

Supplementary file1 (PDF 168 KB)

## Abbreviations

CT: Computed tomography
DOTA-TOC: DOTA(0)-Phe(1)-Tyr(3))octreotide
ER: Experienced reader
ICC: Intraclass correlation coefficient
IR: Inexperienced reader
LN: Lymph node
NET: Neuroendocrine tumor
PET: Positron emission tomography
PRRT: Peptide receptor radionuclide therapy
PSMA: Prostate-specific membrane antigen
RADS: Reporting and Data Systems
SSTR: Somatostatin receptor
TL: Target lesion

## Declarations

### Guarantor

The scientific guarantor of this publication is Johannes Rübenthaler.

### Statistics and biometry

No complex statistical methods were necessary for this paper.

### Methodology

• retrospective

• observational

• performed at one institution

## References

[1] Hofman MS, Lau WF, Hicks RJ (2015). Somatostatin receptor imaging with 68Ga DOTATATE PET/CT: clinical utility, normal patterns, pearls, and pitfalls in interpretation. Radiographics.

[2] Shastry M, Kayani I, Wild D (2010). Distribution pattern of 68Ga-DOTATATE in disease-free patients. Nucl Med Commun.

[3] Werner RA, Solnes LB, Javadi MS (2018). SSTR-RADS Version 1.0 as a reporting system for SSTR PET imaging and selection of potential PRRT candidates: a proposed standardization framework. J Nucl Med.

[4] Bozkurt MF, Virgolini I, Balogova S (2017). Guideline for PET/CT imaging of neuroendocrine neoplasms with (68)Ga-DOTA-conjugated somatostatin receptor targeting peptides and (18)F-DOPA. Eur J Nucl Med Mol Imaging.

[5] Geijer H, Breimer LH (2013). Somatostatin receptor PET/CT in neuroendocrine tumours: update on systematic review and meta-analysis. Eur J Nucl Med Mol Imaging.

[6] Van Essen M, Krenning EP, De Jong M, Valkema R, Kwekkeboom DJ (2007). Peptide receptor radionuclide therapy with radiolabelled somatostatin analogues in patients with somatostatin receptor positive tumours. Acta Oncol.

[7] Zidan L, Iravani A, Kong G, Akhurst T, Michael M, Hicks RJ (2021). Theranostic implications of molecular imaging phenotype of well-differentiated pulmonary carcinoid based on (68)Ga-DOTATATE PET/CT and (18)F-FDG PET/CT. Eur J Nucl Med Mol Imaging.

[8] Strosberg J, El-Haddad G, Wolin E (2017). Phase 3 trial of (177)Lu-dotatate for midgut neuroendocrine tumors. N Engl J Med.

[9] Strosberg J, Wolin E, Chasen B (2018). Health-related quality of life in patients with progressive midgut neuroendocrine tumors treated with (177)Lu-dotatate in the Phase III NETTER-1 Trial. J Clin Oncol.

[10] Kwekkeboom DJ, Kam BL, van Essen M (2010). Somatostatin-receptor-based imaging and therapy of gastroenteropancreatic neuroendocrine tumors. Endocr Relat Cancer.

[11] Krenning EP, Kwekkeboom DJ, Bakker WH (1993). Somatostatin receptor scintigraphy with [111In-DTPA-D-Phe1]- and [123I-Tyr3]-octreotide: the Rotterdam experience with more than 1000 patients. Eur J Nucl Med.

[12] Hicks RJ, Dromain C, de Herder WW (2022). ENETS standardized (synoptic) reporting for molecular imaging studies in neuroendocrine tumours. J Neuroendocrinol.

[13] Weich A, Higuchi T, Bundschuh RA (2022). Training on Reporting and Data System (RADS) for somatostatin-receptor targeted molecular imaging can reduce the test anxiety of inexperienced readers. Mol Imaging Biol.

[14] Werner RA, Derlin T, Rowe SP (2021). High interobserver agreement for the standardized reporting system SSTR-RADS 1.0 on somatostatin receptor PET/CT. J Nucl Med.

[15] Cicchetti D (1994). Guidelines, criteria, and rules of thumb for evaluating normed and standardized assessment instrument in psychology. Psychol Assess.

[16] Diedenhofen B, Musch J (2016). cocron: a web interface and R package for the statistical comparison of Cronbachʼs alpha coefficients. Int J Internet Sci.

[17] Fendler WP, Barrio M, Spick C (2017). 68Ga-DOTATATE PET/CT interobserver agreement for neuroendocrine tumor assessment: results of a prospective study on 50 patients. J Nucl Med.

[18] Werner RA, Weich A, Kircher M (2018). The theranostic promise for neuroendocrine tumors in the late 2010s - where do we stand, where do we go?. Theranostics.

[19] Ambrosini V, Kunikowska J, Baudin E (2021). Consensus on molecular imaging and theranostics in neuroendocrine neoplasms. Eur J Cancer.

[20] Herrmann K, Giovanella L, Santos A (2022). Joint EANM, SNMMI and IAEA enabling guide: how to set up a theranostics centre. J Nucl Med.

[21] Fendler WP, Calais J, Allen-Auerbach M (2017). (68)Ga-PSMA-11 PET/CT interobserver agreement for prostate cancer assessments: an international multicenter prospective study. J Nucl Med.

[22] Woodhouse B, Pattison S, Segelov E et al (2019) Consensus-derived quality performance indicators for neuroendocrine tumour care. J Clin Med 8(9). 10.3390/jcm8091455 10.3390/jcm8091455 PMC6780732 31547431

[23] Kumar R, Sharma P, Garg P (2011). Role of (68)Ga-DOTATOC PET-CT in the diagnosis and staging of pancreatic neuroendocrine tumours. Eur Radiol.

[24] Werner RA, Bundschuh RA, Bundschuh L (2018). Molecular imaging reporting and data systems (MI-RADS): a generalizable framework for targeted radiotracers with theranostic implications. Ann Nucl Med.

[25] Werner RA, Bundschuh RA, Bundschuh L (2019). Novel structured reporting systems for theranostic radiotracers. J Nucl Med.

