# Supplementary material for: Validation of the SSTR-RADS 1.0 for the structured interpretation of SSTR-PET/CT and treatment planning in neuroendocrine tumor (NET) patients
Source: Eur Radiol. 2023 Mar 25;33(5):3416–24. doi: 10.1007/s00330-023-09518-y (PMC10121493; doi:10.1007/s00330-023-09518-y)
Supplement: Supplementary file 1 — Supplementary file1 (PDF 168 KB) [file 330_2023_9518_MOESM1_ESM.pdf]

	Liver	Soft tissue	Lymph node	Skeleton	Lung
**1st read**	42/127 (33.1%)	33/127 (25.9%)	33/127 (25.9%)	15/127 (11.8%)	4/127 (3.2%)
**2nd read**	43/115 (37.4%)	27/115 (23,5%)	30/115 (26.1%)	13/115 (11.3%)	2/115 (1.7%)

**Distribution for overall SSTR-RADS score of all 4 readers**						
**SSTR-RADS score**		**1**	**2**	**3**	**4**	**5**
**1st read**	**ER1**	9	5	2	12	72
	**ER2**	6	1	3	26	64
	**IR1**	0	7	12	12	69
	**IR2**	5	1	2	11	81
**2nd read**	**ER1**	11	5	3	5	76
	**ER2**	7	1	2	22	68
	**IR1**	2	8	7	12	71
	**IR2**	6	3	2	16	73
